# Combined RNA-seq and Phenotype Analysis Reveals a Potential Molecular Mechanism of the Difference in Grain Size of Naked Barley From the Qinghai–Tibetan Plateau

**DOI:** 10.3389/fpls.2022.822607

**Published:** 2022-02-02

**Authors:** Doudou Kong, Jinqing Xu, Lei Wang, Handong Wang, En You, Xiaolan Li, Tongrui Chen, Yuhu Shen

**Affiliations:** ^1^Key Laboratory of Adaptation and Evolution of Plateau Biota, Qinghai Provincial Key Laboratory of Crop Molecular Breeding, Northwest Institute of Plateau Biology, Chinese Academy of Sciences, Xining, China; ^2^Laboratory for Research and Utilization of Qinghai-Tibetan Plateau Germplasm Resources, Northwest Institute of Plateau Biology, Chinese Academy of Sciences, Xining, China; ^3^Innovation Academy for Seed Design, Chinese Academy of Sciences, Xining, China

**Keywords:** barley, RNA-seq, grain size, DEGs, WGCNA

## Abstract

To understand the molecular mechanism controlling the size of barley grains, a number of traits were analyzed and RNA-seq was conducted on grains of two barley materials with a significant difference in thousand-grain weight (TGW) after flowering. The trait dataset delineates the dynamic changes in grain size after flowering, and it provides an understanding of the source of the difference in TGW. By comparing the transcripts of barley grains at several stages after flowering, we identified the gene expression characteristics and significantly enriched pathways in each stage. At the early stage of grain development, genes involved in fatty acid metabolism, plant hormone signal transduction, and pathways involved in cytoskeleton formation were significantly upregulated. At the later stage of grain development, genes involved in starch synthesis, glucose metabolism, and other pathways were significantly upregulated. Further, we used weighted gene coexpression network analysis (WGCNA) and correlation analysis of trait datasets to identify the coexpressed gene modules significantly associated with traits, such as grain length (GL), grain width (GW), and dry weight (DW). After comparing the modules with the differentially expressed gene (DEG) set, 12 candidate genes were selected, and among these, four genes were homologous to genes that regulate grain size in rice and other plants. The combined analysis identified many potential key regulatory factors that may control barley grain size and yield potential, thus providing new insights into the molecular mechanism of barley grain size.

## Introduction

Crop breeding programs are facing serious challenges. Rapid population growth and urbanization have gradually reduced the arable land area. This means that all crop yields will need to increase dramatically in the coming decades. In general, yield per plant is determined by panicle number, grain number per panicle, and grain weight per panicle, which are the result of the joint action of multiple genes (Wilhelm et al., 2013). However, the variability in grain weight of a single genotype makes it difficult to identify traits, as genetic mapping relies on a clear distinction in phenotypes. Recent studies have separated grain weight into multiple subcomponents, such as grain width (GW), grain length (GL), and grain thickness, some of which have been shown to be controlled by independent genetic pathways (Brinton and Uauy, 2019).

The Qinghai–Tibetan Plateau is the world’s largest high-altitude ecosystem, with an average altitude exceeding 4,000 m. The high altitude makes the habitat of the Qinghai–Tibet Plateau very harsh, and plants growing in this area must be adapted to the extreme environment with low temperature, low oxygen, and strong UV light. Naked barley is the staple crop in this area, owing to traits, such as early maturity, cold and drought tolerance, and short growing time. In contemporary society, where food safety and quality are sought, health foods derived from barley have gradually become more common in everyday life. It is predicted that the current yield of barley will not be able to meet the continuously increasing market demand, and yield remains the most important target in barley breeding. Thus, understanding the genetic mechanisms that maintain high yields under adverse conditions at high altitudes is crucial to improving crops in marginal environments (Xia et al., 2017).

Compared with the mechanisms in plants, such as *Arabidopsis* and rice, which have been studied extensively, the understanding of the barley grain size formation mechanism is still very limited. Zhou et al. (2016) mapped two main quantitative trait loci (QTLs) for barley GL in a recombinant inbred line (RIL) population. Walker et al. (2013) identified 11 grain traits and 232 QTLs in double-haploid (DH) populations grown in three environments. Wendt et al. (2016) discovered a dep1 loss-of-function mutation in the Golden Promise cultivar. The *HvDep1* gene encodes the AGG3 subunit, which positively regulates barley stem elongation and grain size. In the most recent study by Watt et al. (2020), 306 DH populations were constructed through the hybridization of Vlamingh and Buloke, two cultivars with significant differences in grain length, and a main QTL for barley grain length, *qGL2H*, was precisely mapped. Within this new localization interval, the researchers found three predictive genes with high confidence, one of which encodes an MYB transcription factor that is associated with biological functions, such as cell division, grain length regulation, and abiotic stress tolerance (Watt et al., 2020). This provides a promising candidate site for further study of gene function.

In this study, we performed detailed RNA-seq analysis on the grains of two barley species with different grain size phenotypes at different days after flowering (DAF). The dataset of the early stages of grain development provides a comprehensive and system-level view of dynamic gene expression networks and their potential role in controlling grain size. Using pairwise comparison and weighted gene coexpression network analysis (WGCNA), we identified coexpressed gene modules and 12 candidate genes for grains at each developmental stage after flowering. The relative expression levels of the 12 genes were confirmed to be consistent with the RNA-seq results. Four of these genes are homologous to known grain size-regulating genes in rice and other plants. This work provides important insights into the molecular network of barley grain development.

## Materials and Methods

### Plant Materials

From 853 barley materials collected in our laboratory, two six-rowed naked barley with a significant difference in thousand-grain weight (TGW) were selected, namely, large-grain material (LG, breeding variety Beiqing 3, TGW = 55.93 g) and small-grain material (SG, breeding Line 3,917, TGW = 34.20 g). They were planted at the experimental base of the Northwest Institute of Plateau Biology, Chinese Academy of Sciences (N36°30′, E101°33′, altitude is 2,750 m) in Haimaquan Village, Lushaer Town, Huangzhong County, Qinghai Province from 2017 to 2019.

### Trait Measurements

When the first anthers of the main spike were exposed to the glumes, the main spike was marked; this was recorded as 0 days after flowering (0 DAF). Then, at 2 DAF, 4 DAF, 6 DAF, 8 DAF, 10 DAF, 15 DAF, 20 DAF, 22 DAF, 25 DAF, 30 DAF, and 35 DAF (11 different stages), 15 single spikes were collected, and the grains of eight spikelets in the middle of each spike were taken for trait measurements. Three biological replicates were performed. The fresh weight (FW) of the grains was measured by balance. Then, the fresh grains were dried in an oven at 75°C for 72 h, and the dry weight (DW) was measured. The “water displacement” principle was used to measure the grain volume (Yan et al., 2018). The grain length and GW were measured using a Vernier caliper, and the grain area was measured by ImageJ.

### RNA Extraction and Transcriptome Sequencing

Previous studies have divided the development of Triticeae grains into three stages: cell division and expansion (0–14 DAF), grain filling (14–28 DAF), and grain maturation and dehydration (from 28 DAF). It is considered that the changes in grain length, grain width, and DW occur mainly during the grain filling stage (Brinton and Uauy, 2019). As references, we chose barley grains at 4 DAF, 8 DAF, 15 DAF, and 22 DAF for RNA-seq, among which 4 and 8 DAF represented active stages of cell division and amplification, while 15 and 22 DAF represented the grain filling stage. Five main spikes were taken from each stage, and one grain from the 1/3 of the spike closest to the base was selected from each plant and mixed with each other; three replicates were performed for each stage. Total RNA was extracted from grains using TRIzol reagent (Invitrogen, United States). RNA concentration was measured using a NanoDrop 2000 spectrophotometer (Thermo Fisher Scientific, Inc., United States) after DNase treatment, and RNA integrity was measured using an Agilent 2100 bioanalyzer (Agilent Technologies, Inc., United States). The cDNA library was constructed with 3 μg RNA and sequenced by the Illumina high-throughput sequencing platform.

### RNA-seq Reads Alignment and Expression Analysis

Raw data were obtained from the Illumina high-throughput sequencing platform, and clean data were generated by removing the reads containing adaptor sequences, more than 5% ambiguous bases (noted as N) and 50% low-quality bases (Phred quality score Q < 30). The raw sequencing data were deposited into the NCBI SRA database with accession number SRP344987. The high-quality reads were aligned to the barley reference genome (Mascher et al., 2017) using Hisat2 (Kim et al., 2015). The transcripts were assembled and merged under GFF annotation using the StringTie merge function to create a common set of transcripts for all libraries (Pertea et al., 2015). Furthermore, DESeq2 was used for differential expression analysis between groups using a statistical model based on the negative binomial distribution to calculate the fold change in differential expression (fold change) and significance (*p* value) of genes. Genes with a fold change greater than 2 and a false discovery rate (FDR, Benjamini and Hochberg’s method) less than 0.01 (Anders and Huber, 2010; Love et al., 2014) were considered differentially expressed genes (DEGs). All DEGs were mapped to GO terms in the Gene Ontology database,^1^ and KOBAS 2.0 (Xie et al., 2011) was used to obtain KEGG Orthology results. GO enrichment and KEGG pathway analysis were performed using the TBtools toolkit (Chen et al., 2020). The results were visualized using ggplot2 and PheatMap R packages.

### Construction of a Weighted Gene Coexpression Network and Visualization of Gene Interaction Network

The WGCNA (Langfelder and Horvath, 2008) method was used to construct a gene coexpression network for all samples of the two materials in four stages. The concrete operation is built with the “one-step method” of the “WGCNA” package in R 3.4.3 software. FPKM > 1 was selected as the standard for screening the genes. To make the gene network conform to the characteristics of non-scale distribution, a soft threshold = 6 was selected, which could construct a network close to the 85% degree of freedom. Different genes were classified into different modules by the dynamic tree cut method, and the minimum number of genes in each module was set to 30. The shearing height was set to 0.2, and the coexpression modules with similar clustering were merged. In addition, the correlation between the module and the characters was calculated. Furthermore, the module membership (MM) and the gene significance (GS) correlation coefficient of genes and traits in a specific module were calculated, with the MM value and GS value used as horizontal and vertical coordinates of scatterplots. The 500 connection relationship matrices with the highest gene connectivity in the module were exported and used to construct and visualize the gene network diagram in Cytoscape 3.7.1. The genes with the highest connectivity in the center of the network are called hub genes and were considered candidate genes to be highly related to grain development at different stages. The top 20 genes related to the trait (using the GS value as the sorting criterion) and the DEGs were intersected, and then, the connection relationships between the intersecting genes and the genes in the module were exported and visualized as a gene network diagram by Cytoscape 3.7.1. Genes that are located in the center of the network and have higher correlations are considered candidate genes.

### Quantitative Reverse Transcription PCR Analysis

According to the manufacturer’s protocols, the total RNA (1 μg) in each sample was used to generate cDNA templates using the PrimeScript™ RT reagent Kit with gDNA Eraser (Takara, Japan) for qPCR. The TB Green™ *Premix Ex Taq*™ II kit (Takara, Japan) was used to configure the reaction solution, and the ABI 7500 Fast Real-Time PCR System (Applied Biosystems, United States) was used to perform the quantitative reverse transcription PCR (qRT-PCR). The PCR conditions included predenaturation at 95°C for 30 s, followed by 40 cycles of 95°C for 15 and 60°C for 34 s. The specific primer pairs used for qRT-PCR are listed in Supplementary Table 1. For each sample, the PCR analysis was repeated independently in triplicate, and the quantification of gene expression was carried out by the relative quantification method (2^-ΔΔCT^ method). *α*-Tubulin served as an endogenous control.

## Results

### Dynamic Changes of Grains After Flowering

**Figure 1:**
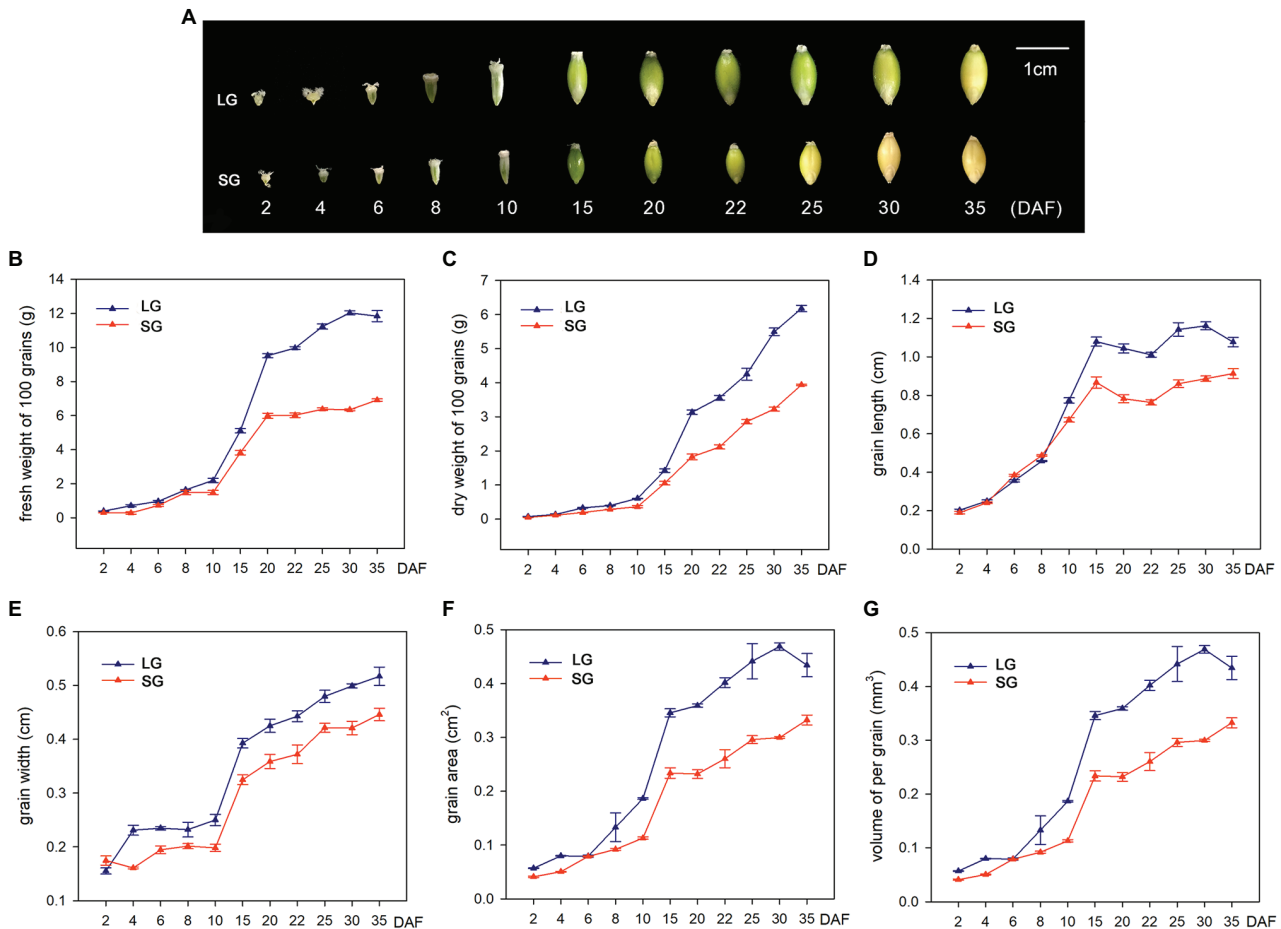
**(A)** Dynamic changes of grain morphology of large-grain material (LG) and small-grain material (SG) in different stages after flowering. **(B–G)** Dynamic changes of six traits of LG and SG at 11 stages: **(B)** 100-grain fresh weight (FW), **(C)** 100-grain dry weight (DW), **(D)** grain length (GL), **(E)** grain width (GW), **(F)** grain area, and **(G)** volume of per grain.

In order to explore the potential mechanisms underlying the grain size and weight differences between LG and SG, we first observed the grain morphology of two barley materials at different stages after flowering. The results showed that the grains expanded rapidly after flowering, especially from 10 DAF to 20 DAF, and the grain length, grain width, and grain area increased most significantly. Before 15 DAF, the grain morphology was basically conical and then gradually expanded to a spindle shape. At 20 DAF, the grain morphology was essentially finalized, and the grain length, grain width, and grain area entered the plateau growth stage. In the later stages of development, dehydration reduces the grain size before full maturation (Figure 1A). The FW of the two barley materials showed slow growth before 10 DAF, demonstrated exponential growth between 10 DAF and 20 DAF and slowed in growth after 20 DAF. Before 30 DAF, the fresh weight of LG material was continuously higher than that of SG. After 30 DAF, the fresh weight decreased, but the dry weight continued to increase, indicating that LG material not only had an advantage over SG in grain weight but also had a faster rate of dry matter accumulation than SG. Similar to the growth trend of fresh weight, dry weight showed flat, slow growth before 10 DAF and exponential growth after 10 DAF, basically maintaining a high growth rate. This indicates that grain filling begins at approximately 10 DAF, and the dry weight increases continuously. The dry weight of LG continued to be higher than that of SG after 10 DAF (Figures 1B,C). In the dynamic change curve of grain length and grain width, LG maintains a longer grain length from 10 DAF and maintains a larger grain width from 4 DAF (Figures 1D,E). The grain area is determined by the grain length and grain width, which reflects the joint effect of the two in the formation of grain morphology. The grain area increased slowly before 10 DAF, increased rapidly from 10 DAF to 15 DAF, and then increased slowly thereafter (Figure 1F). The volume of LG grains showed an overall increase and was significantly greater than SG (Figure 1G).

### Gene Expression Profiles at Different Stages After Flowering

Transcriptome sequencing and transcriptome profiling were performed on the grain samples at different stages after flowering. After sequencing quality control, the percentage of Q30 bases of each sample was not less than 89.49%, indicating that they were suitable for further analysis (Supplementary Table 2). Principal component analysis (PCA) and cluster analysis were performed on 24 samples of the two materials to identify the differences in expression characteristics between samples and whether there were outliers (Supplementary Figure 1). PCA and cluster analysis showed that the expression patterns at 4 DAF and 8 DAF in the cell division and expansion stages of the two materials were relatively concentrated, and the differentiation of cluster trees was relatively close together. In contrast, the PCA space distances of 15 DAF and 22 DAF were relatively far apart. The cluster trees demonstrated obvious differentiation, suggesting significant differentiation of gene expression patterns within LG and SG at the filling stage (starting from 15 DAF; Supplementary Figure 1).

**Figure 2:**
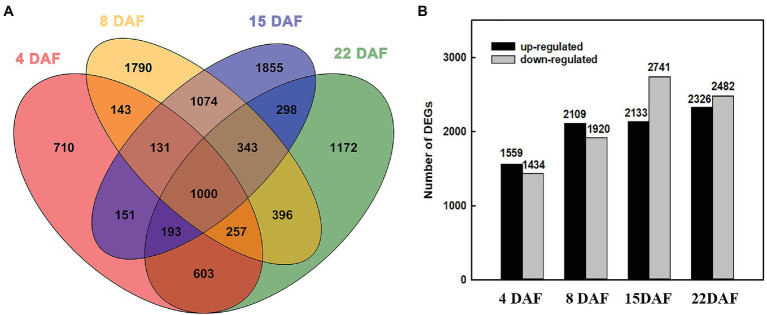
Differentially expressed genes (DEGs) at different stages. **(A)** Venn diagram of DEGs among the grains of two barley materials. **(B)** The number of DEGs between large-grain material (LG) and small-grain material (SG) at different stages.

To characterize the transcriptional changes between the large and small grains, we investigated the DEGs of the two materials at different stages. At 4 DAF, 1,559 genes were upregulated in LG, and 1,434 genes were downregulated (Supplementary Table 3). At 8 DAF, 2,109 genes were upregulated, and 1,920 genes were downregulated (Supplementary Table 4). At 15 DAF, 2,133 genes were upregulated, and 2,741 genes were downregulated (Supplementary Table 5). At 22 DAF, 2,326 genes were upregulated, and 2,482 genes were downregulated in LG (Supplementary Table 6). From 4 DAF to 22 DAF, the number of upregulated and downregulated genes increased gradually, indicating that physiological activity was more abundant at the grain filling stage than at the cell division stage (Figure 2).

**Figure 3:**
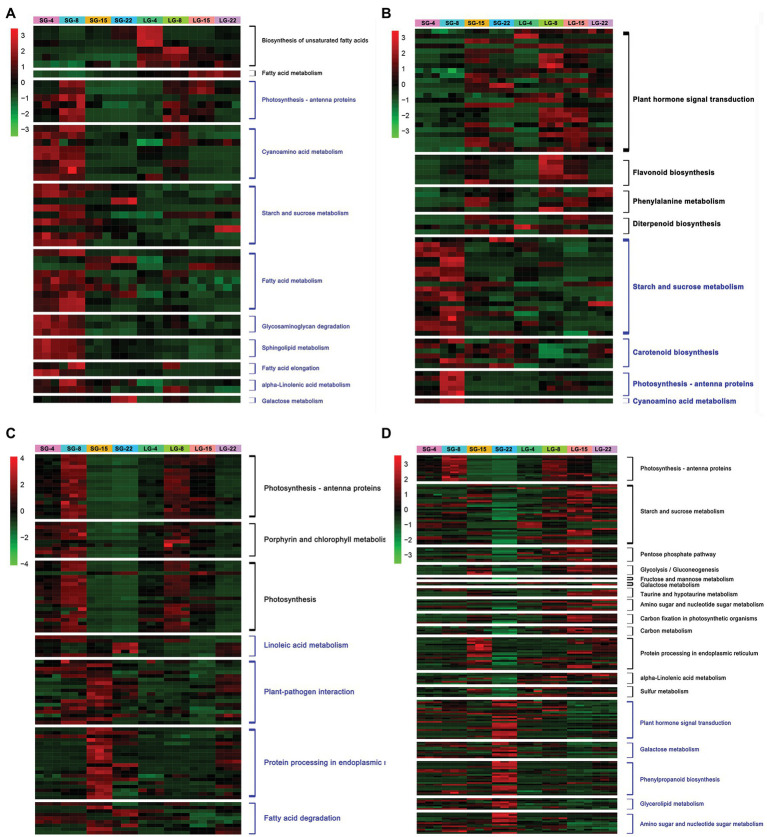
Heatmap of differentially expressed genes (DEGs) expression at each stage. **(A)** DEGs significantly up/downregulated during 4 days after flowering (DAF). **(B)** DEGs significantly up/downregulated during 8 DAF. **(C)** DEGs significantly up/downregulated during 15 DAF. **(D)** DEGs significantly up/downregulated during 22 DAF. The black font on the right side of the heatmap indicates the pathways enriched by DEGs upregulated in LG, and blue font indicates the pathways enriched by DEGs downregulated in LG.

Enrichment analysis was conducted on the upregulated and downregulated genes at each stage. The metabolic pathways significantly upregulated at 4 DAF in LG were mainly the biosynthesis of unsaturated fatty acids and fatty acid metabolism. The expression of HORVU3Hr1G061520 and HORVU3Hr1G092740, encoding fatty acid desaturase, was increased by more than 10 times in LG. The significantly downregulated genes in LG at 4 DAF included photosynthesis–antenna protein, cyanoamino acid metabolism, starch and sucrose metabolism, fatty acid metabolism, glycosaminoglycan degradation, and sphingolipid metabolism, among others. Among these, the expression of HORVU3Hr1G033160, which encodes pectinesterase, was downregulated more than 15-fold in LG (Figure 3A; Supplementary Table 7).

The pathways that were significantly upregulated in LG at 8 DAF included plant hormone signal transduction, flavonoid biosynthesis, phenylalanine metabolism, and diterpene biosynthesis, among which a total of 25 DEGs were enriched in the plant hormone signal transduction pathway. HORVU5Hr1G050270 encodes an unknown cyclin that is involved in physiological activities, such as cell division and the cell cycle. Upregulation of this gene indicates that cell division and other activities are more vigorous in LG. HORVU4Hr1G016110 encodes an AUX/IAA family auxin response factor, and the expression level of this gene was over seven times greater in LG than in SG. Previous studies have shown that the AUX-responsive factor *tgw6* in the AUX/IAA family in rice can affect the transition from the syncytial phase to the cellularization phase by controlling the supply of IAA, thus limiting the number of cells and the length of grains (Ishimaru et al., 2013). The pathways in which 8 DAF was significantly downregulated in LG included starch and sucrose metabolism, carotenoid biosynthesis, photosynthesis–antenna protein, and cyanoamino acid metabolism. A total of 20 DEGs were enriched in the starch and sucrose metabolic pathways, including genes encoding glycosyl hydrolase and sucrose synthase, which were significantly downregulated (Figure 3B; Supplementary Table 7).

The pathways that were upregulated in LG at 15 DAF included photosynthesis–antenna protein, photosynthesis, and porphyrin and chlorophyll metabolism. Categories that were downregulated included linoleic acid metabolism, plant–pathogen interaction, protein processing in the endoplasmic reticulum, and fatty acid degradation. Fourteen genes encoding heat shock proteins (Hsps) were significantly downregulated, among which eight genes were downregulated by more than 20 times in LG. However, no Hsp family proteins have been reported to be involved in grain size regulation (Figure 3C; Supplementary Table 7).

At 22 DAF, there were 13 enrichment pathways that were significantly upregulated in LG, including photosynthesis–antenna protein, starch and sucrose metabolism, pentose phosphate pathway, glycolysis/gluconeogenesis, and fructose and mannose metabolism. The enriched starch and sucrose metabolic pathways were represented by 37 genes, most of which encoded glycosyl hydrolases, amylases, and hexokinases, and their expression levels were increased by 2–12 times. Starch and glucose metabolism comprise a variety of biochemical processes responsible for carbohydrate formation, decomposition, and mutual transformation (Yan et al., 2018). Sucrose metabolism provides materials and energy sources for cell wall and starch biosynthesis. The DEGs showed significant enrichment in sucrose degradation, including sucrose synthase and invertase genes. Pathways that were significantly downregulated at 22 DAF included plant hormone signal transduction, galactose metabolism, phenylpropanoid biosynthesis, glycerol metabolism, and amino sugar and nucleotide sugar metabolism (Figure 3D; Supplementary Table 7).

Comparing the significantly enriched pathways of DEGs in the four stages, the number of upregulated pathways increased and the number of downregulated pathways decreased over time. Genes for physiological processes, such as phytohormone signal transduction, are upregulated in LG in the early stage of grain development. This may be due to the rapid growth of early grains and vigorous physiological activities, such as cell division. In later grain development, genes involved in physiological processes, such as starch synthesis, glucose metabolism, and carbon metabolism, which are related to dry matter accumulation in the endosperm, were significantly upregulated in LG (Figure 3).

### Weighted Gene Coexpression Network Analysis

Weighted gene coexpression network analysis was performed to understand the correlation between the sample expression profile and the sample phenotype. In this study, a total of 25,455 genes were expressed in the samples (Supplementary Table 8), and after filtering out the low-expression genes (FPKM < 1), 9,222 genes were used in the construction of the coexpression network. To meet the premise of the scale-free network distribution, the soft threshold *β* of the adjacency matrix needs to be determined. The correlation coefficient of gene expression between 1 and 30 and the average connection value of adjacent genes were calculated. According to the simulation results of *β* = 1–30, the gene expression correlation coefficient and the average connection value curve are close to 1 (i.e., an inflection point appears) when *β* = 6; thus, *β* = 6 was selected to construct the gene network (Supplementary Figure 2).

**Figure 4:**
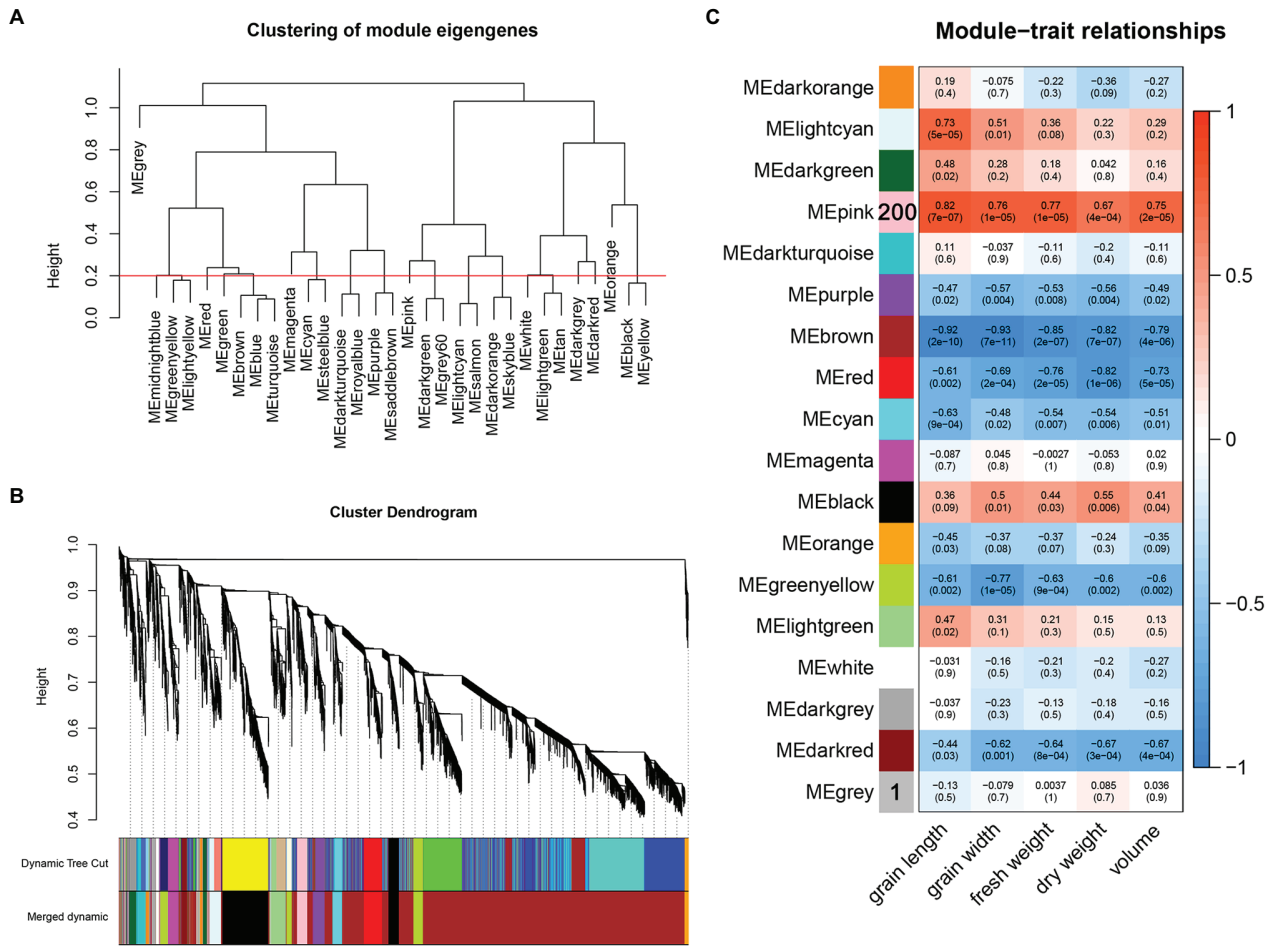
**(A)** Clustering of module eigengenes and height, the red line represents the shearing height. Modules below the red line have similar expression patterns and are merged into one module. **(B)** Gene cluster tree after merging. **(C)** Correlation between modules and traits.

After converting the filtered FPKM into a topological overlap matrix (TOM), we performed hierarchical cluster analysis on each gene and classified different genes into coexpression modules after dynamic cutting. The minimum number of genes in each module was set to 30. Modules with shear heights below 0.2 were combined into one module (Figures 4A,B), and the correlation between different modules was calculated using grain length, grain width, fresh weight, dry weight, and volume as the trait matrix. Finally, a total of 17 modules were obtained (Figure 4C). Among these 17 modules, the pink module was significantly related to grain length (*r* = 0.82, *p* = 7e−07), grain width (*r* = 0.76, *p* = 1e−05), fresh weight (*r* = 0.77, *p* = 1e−05), dry weight (*r* = 0.67, *p* = 4e−04), and volume (*r* = 0.75, *p* = 2e−05).

### The Pink Module Contains Genes Involved in Physiological Activities

**Figure 5:**
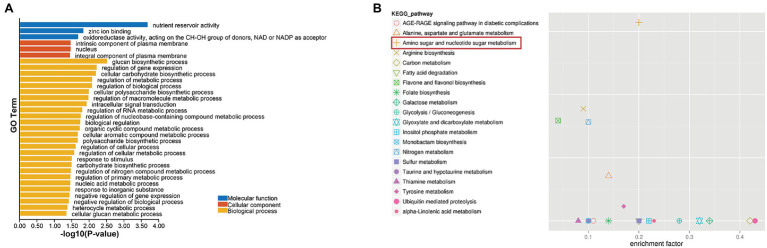
GO annotation **(A)** and KEGG pathway enrichment **(B)** results of the pink module genes.

The pink module contains a total of 200 genes. GO annotation and KEGG pathway analysis were performed on the genes in the module. The most significantly enriched GO annotation term was nutrient reservoir activity in molecular function and glucan biosynthetic process in biological process. The most significantly enriched KEGG pathway was amino sugar and nucleotide sugar metabolism, indicating that sugar metabolism was significantly related to selected traits (Figure 5; Supplementary Tables 9, 10).

**Figure 6:**
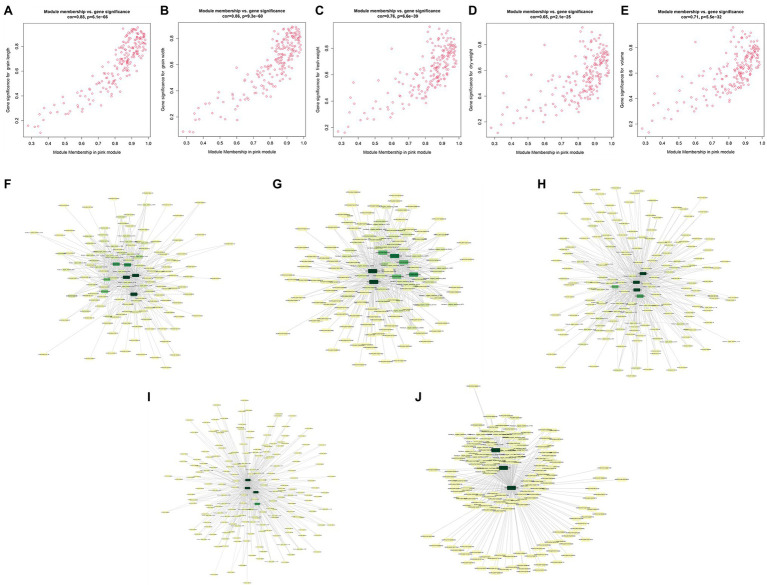
Module membership (MM)-gene significance (GS) correlation scatter plot **(A)** grain length (GL); **(B)** grain width (GW); **(C)** fresh weight (FW); **(D)** dry weight (DW); and **(E)** volume (V). Intersection gene network diagram **(F)** GL; **(G)** GW; **(H)** FW; **(I)** DW; and **(J)** volume.

Module membership and GS in a module reflect the correlation between genes in a module and sample traits (Supplementary Table 11). We drew a pink module MM-GS scatter diagram (Figure 6). The top 20 genes associated with traits (with GS value as the sorting criterion) and the DEGs were intersected, and then, the intersecting genes were combined with the module. Then, the connection relationship between the intersecting genes and the genes in the module was exported, and the gene network map was obtained (Figure 6). Genes located in the center of the network with high correlation were selected as candidate genes. Since the five traits were all significantly related to the pink module, after the removal of duplicate genes, 12 candidate genes remained (Table 1).

**Table 1:** List of candidate genes.

Gene ID	Description
HORVU6Hr1G055960	Bidirectional sugar transporter
HORVU1Hr1G061160	Pyruvate, phosphate dikinase 2
HORVU7Hr1G020860	Early nodulin-93-like
HORVU7Hr1G020880	Early nodulin-93-like
HORVU0Hr1G029210	Early nodulin-93-like
HORVU1Hr1G088510	Mitogen-activated protein kinase
HORVU5Hr1G080540	E3 Ubiquitin ligase BIG BROTHER
HORVU7Hr1G006280	b-Type farinin protein
HORVU1Hr1G000680	Putative gamma 2 hordein
HORVU6Hr1G001150	Alpha-amylase inhibitor BDAI-1
HORVU1Hr1G005030	C Hordein
HORVU2Hr1G126740	Chitinase 2

The 12 selected candidate genes were involved in intercellular sugar transport, PEP utilization enzymes, nodulin family proteins, MAPK signaling, E3 ubiquitin ligase, hordein, α-amylase inhibitors, and chitinase. Several pathways have been proven to participate or indirectly participate in the regulation of grain size. HORVU6Hr1G055960 is annotated to the SWEET family intercellular sugar transporter, which is significantly upregulated in LG at 8 DAF and 15 DAF. HORVU1Hr1G061160 encodes pyruvate phosphodikinase (PPDK), which participates in the transport and metabolism of carbohydrates and is significantly related to grain length. It was significantly upregulated in LG at 8 DAF and 15 DAF. HORVU7Hr1G020860, HORVU7Hr1G020880, and HORVU0Hr1G029210 encode early Nodulin-93-like (ENODs). Among them, HORVU7Hr1G020860 was significantly upregulated in LG at all four stages. HORVU7Hr1G020880 and HORVU0Hr1G029210 were significantly upregulated at 8 DAF, 15 DAF, and 22 DAF. HORVU1Hr1G088510 encodes a member of the MPK family of mitogen-activated protein kinases and was significantly upregulated in LG at 8 DAF and 15 DAF. HORVU5Hr1G080540 encodes an E3 ubiquitin ligase, which is an ortholog of the E3 ubiquitin ligase BIG BROTHER (BB) in wheat. It was significantly upregulated in LG at 8 DAF and 15 DAF. HORVU7Hr1G006280 encodes a b-type farinin protein, which is significantly upregulated in LG at 15 DAF and 22 DAF. HORVU1Hr1G000680 encodes γ-2 gliadin and was significantly upregulated in LG at 4 DAF, 8 DAF, and 15 DAF. HORVU6Hr1G001150 encodes the α-amylase inhibitor BDAI-1 and was significantly upregulated in LG at 4 DAF, 8 DAF, and 15 DAF. HORVU1Hr1G005030 encodes a type C hordein and was significantly upregulated in LG at 8 DAF and 15 DAF. HORVU2Hr1G126740 encodes chitinase and was significantly upregulated in LG at 8 DAF, 15 DAF, and 22 DAF.

### Gene Expression Verification by qRT-PCR

**Figure 7:**
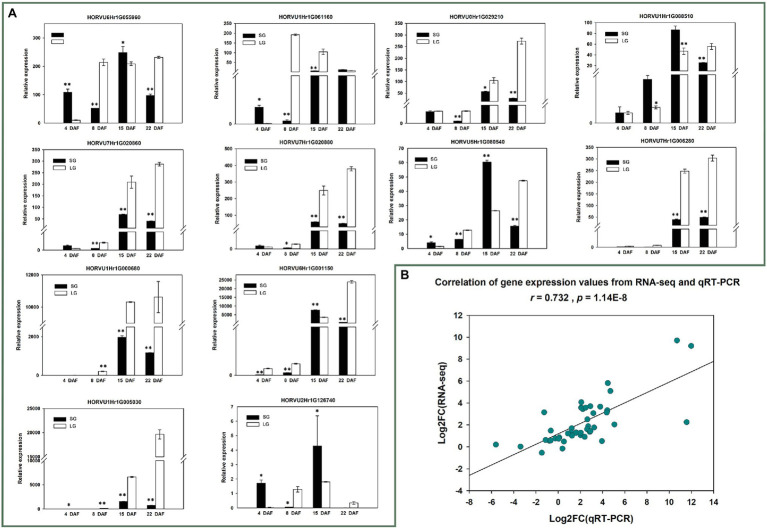
**(A)** Quantitative reverse transcription PCR (qRT-PCR) relative expression of 12 candidate genes; ^*^p < 0.05; ^**^p < 0.01. **(B)** Correlation between RNA-seq gene expression and qRT-PCR relative expression.

To further verify the reliability of the RNA-seq data and the expression patterns of 12 candidate genes, qRT-PCR was conducted for the 12 genes in materials from all four stages. The verification results of 24 samples were basically consistent with the RNA-seq results (*r* = 0.732, *p* = 1.1362E−8; Figure 7; Supplementary Table 12).

Furthermore, we calculated the correlations between the difference of expression levels of 12 candidate genes and the difference of phenotypic traits in different stage (Table 2). The results showed that at 4 DAF, HORVU1Hr1G000680, HORVU1Hr1G005030, and HORVU6Hr1G001150 were significantly positive correlated with the grain width. HORVU1Hr1G000680 and HORVU1Hr1G005030 were significantly positive correlated with the fresh weight, while the HORVU1Hr1G088510 were significantly negative correlated with the fresh weight. At 8 DAF, almost all the candidate genes were significantly negative with the grain length, while all the candidate genes were significantly positive correlated with the dry weight. At 15 and 22 DAF, almost all the candidate genes were significantly positive correlated with the phenotypic traits. The results indicated that, compared with the cell division and amplification stage, the candidate genes were more likely function in the grain filling stage, which result the grain size difference of the two materials.

**Table 2:** List of candidate genes.

		HORVU0Hr1G029210	HORVU1Hr1G000680	HORVU1Hr1G005030	HORVU1Hr1G061160	HORVU1Hr1G088510	HORVU2Hr1G126740	HORVU5Hr1G080540	HORVU6Hr1G001150	HORVU6Hr1G055960	HORVU7Hr1G006280	HORVU7Hr1G020860	HORVU7Hr1G020880
4 DAF	GL	0.38	0.45	0.46	−0.06	−0.31	−0.33	0.4	0.44	−0.43	0.72	−0.2	0.3
	GW	0.47	0.99^**^	0.99^**^	0.26	−0.69	0.28	−0.44	0.98^**^	−0.66	0.90^*^	−0.43	0.42
	V	0.79	0.51	0.45	−0.16	−0.11	−0.04	−0.08	0.53	−0.14	0.62	−0.69	0.77
	FW	0.35	0.90^**^	0.91^**^	0.29	−0.94^**^	0.05	−0.01	0.87^*^	−0.88^*^	0.86^*^	−0.37	0.33
	DW	0.46	0.45	0.37	0.35	−0.57	−0.37	0.18	0.49	−0.61	0.42	−0.58	0.57
8 DAF	GL	−0.94^**^	−0.95^**^	−0.96^**^	−0.95^**^	−0.93^**^	−0.88^**^	−0.97^**^	−0.91^**^	−0.96^**^	−0.73	−0.94^**^	−0.95^**^
	GW	0.66	0.74	0.74	0.71	0.86^*^	0.66	0.76	0.51	0.75	0.77	0.68	0.75
	V	0.53	0.57	0.56	0.56	0.57	0.54	0.56	0.47	0.59	0.57	0.55	0.58
	FW	0.52	0.53	0.54	0.51	0.56	0.49	0.61	0.46	0.56	0.49	0.53	0.54
	DW	0.96^**^	0.98^**^	0.99^**^	0.98^**^	0.98^**^	0.93^**^	0.98^**^	0.91^**^	0.99^**^	0.82^**^	0.96^**^	0.99^**^
15 DAF	GL	0.92^*^	0.90^*^	0.92^**^	0.96^**^	0.92^**^	0.95^**^	0.91^*^	0.90^*^	0.94^*^	0.96^*^	0.93^*^	0.94^*^
	GW	0.93^**^	0.94^**^	0.96^**^	0.95^**^	0.90^*^	0.94^**^	0.88^*^	0.90^*^	0.92^**^	0.92^**^	0.92^**^	0.92^**^
	V	0.99^**^	0.99^**^	0.99^**^	0.98^**^	0.88^*^	0.99^**^	0.93^**^	0.97^**^	0.98^**^	0.98^**^	0.99^**^	0.98^**^
	FW	0.93^**^	0.96^**^	0.97^**^	0.98^**^	0.90^*^	0.96^**^	0.87^*^	0.91^*^	0.95^**^	0.97^**^	0.94^**^	0.94^**^
	DW	0.88^*^	0.90^*^	0.93^**^	0.97^**^	0.99^**^	0.93^**^	0.82^*^	0.85^*^	0.90^*^	0.94^**^	0.91^*^	0.90^*^
22 DAF	GL	0.95^*^	0.99^**^	0.99^**^	0.99^**^	0.97^**^	0.99^**^	0.99^**^	0.98^**^	0.97^**^	0.99^**^	0.99^**^	0.98^**^
	GW	0.8	0.86^*^	0.87^*^	0.87^*^	0.81	0.86^*^	0.83^*^	0.87^*^	0.88^*^	0.84^*^	0.91^*^	0.88^*^
	V	0.94^**^	0.92^**^	0.92^**^	0.94^**^	0.90^*^	0.94^**^	0.90^**^	0.95^**^	0.97^**^	0.92^*^	0.96^**^	0.96^**^
	FW	0.97^**^	0.99^**^	0.99^**^	0.99^**^	0.97^**^	0.99^**^	0.97^**^	0.99^**^	0.99^**^	0.99^**^	0.99^**^	0.99^**^
	DW	0.96^**^	0.99^**^	0.99^**^	0.99^**^	0.98^**^	0.99^**^	0.99^**^	0.99^**^	0.98^**^	0.99^**^	0.99^**^	0.98^**^

GL, grain length; GW, grain width; V, volume; FW, fresh weight; and DW, dry weight.

** Correlation is significant at the 0.01 level (two-tailed).

* Correlation is significant at the 0.05 level (two-tailed).

## Discussion

Comparing the expression levels of the homologs of genes controlling grain size among different species and revealing previously unknown gene expression patterns is an effective method to explore the molecular mechanism of grain size. In the current study, barley grains at 4 DAF, 8 DAF, 15 DAF, and 22 DAF were selected for transcriptome sequencing, and DEGs between large and small grains at each stage were obtained. We tested the differential expression of the barley homologs of genes known to control grain size genes in other plants in four stages and found that only a few genes showed significant differential expression levels between large- and small-grain materials. At 4 DAF, the expression of HORVU3Hr1G068000, the homolog of *BRI1*, was significantly downregulated in LG. *bri1* is a BR-insensitive mutant, and grain length increases after applying BR (Jiang et al., 2013). The barley genes HORVU6Hr1G068370, HORVU6Hr1G077840, and HORVU4Hr1G065440, homologs of *GS2*, *GAD1*, and *DWF4*, respectively, were significantly upregulated in LG. *GRAIN SIZE 2* (*GS2*) encodes growth-regulating factor 4 (OsGRF4), and OsGRF4 is regulated by microRNA396 (OsmiR396). The *GS2^AA^* allele of OsGRF4, which is mutated at the target site of OsmiR396, destroyed the inhibitory effect of OsmiR396 on OsGRF4, resulting in the increased expression of OsGRF4 and subsequently increased grain length and grain weight (Duan et al., 2016; Li and Li, 2016). The upregulation of *GS2* homolog expression was detected in LG, indicating that HORVU6Hr1G068370 may also play a role in increasing grain length. *GAD1* encodes a small signal peptide that promotes cell division at the top of the glume shell to increase grain elongation (Jin et al., 2016). *DWF4* encodes a BR biosynthesis enzyme, and its overexpression can increase grain size (Jiang et al., 2013). Its homolog in barley, HORVU4Hr1G065440, was upregulated in LG, which is speculated to be one of the reasons for the emergence of the large-grain phenotype.

At 8 DAF, the *BB* homolog HORVU5Hr1G080540 was significantly upregulated in LG. The *GW5* homolog HORVU1Hr1G028250 and the *GW8* homolog HORVU0Hr1G039170 were significantly downregulated in LG. HORVU5Hr1G080540 encodes an E3 ubiquitin ligase, which is an ortholog of the E3 ubiquitin ligase BB. In rice and wheat, the *BB* genes controlled the grain size by limiting the duration of cell proliferation (Disch et al., 2006; Xia et al., 2013). *GW5* is a major QTL that controls grain width, and grain width increases after *GW5* deletion (Weng et al., 2008). *GW8* encodes the transcription factor OsSPL16, which contains an SBP domain. Overexpression of *GW8* can lead to increased grain width, and knockout of *GW8* can narrow the grain (Lee et al., 2006; Wang et al., 2015a,b). Considering that HORVU0Hr1G039170 is the homolog of *GW8*, it is speculated that HORVU0Hr1G039170 is involved in the regulation of grain width in LG.

At 15 DAF, the *BB* homolog HORVU5Hr1G080540 was significantly upregulated in LG, while the *OsBZR1* homolog HORVU3Hr1G026420 was significantly downregulated. *OsBZR1* is a downstream molecule in the brassinolide signaling pathway. Overexpression of *OsBZR1* increases sugar accumulation, grain length, grain width, TGW, and spikelet number in grains, while its knockout results in smaller grains, decreased TGW, and starch accumulation (Jiang et al., 2013). Interestingly, HORVU3Hr1G026420, the homolog of *OsBZR1*, was significantly downregulated in 56, but the phenotype of smaller grains did not appear, indicating that the function of this gene in rice and barley is different.

At 22 DAF, the *GS5* homolog HORVU3Hr1G033550 was significantly upregulated in LG, while the *GW5* homolog HORVU1Hr1G028250 was significantly downregulated. *GS5* encodes an unknown serine carboxylpeptidase, and overexpression of *GS5* can accelerate glume cell proliferation, which is reflected in an increase in grain width and weight (Li et al., 2011). The function of the homologous protein in maize, ZmGS5, is the same as that in rice (Liu et al., 2015). Combined with the significant upregulation of the *GS5* homolog in LG, it is speculated that HORVU3Hr1G033550 is also involved in the formation of the big grain phenotype. At 8 DAF, the *GW5* homolog was also significantly downregulated. The loss of *GW5* function leads to a failure of ubiquitin transfer to its target protein, so that the substrate that should be degraded cannot be specifically identified, thereby activating the division of glume cells and ultimately increasing the glume width and grain weight (Weng et al., 2008).

Through differential gene analysis and pathway enrichment of gene expression in a specific stage, it was found that as grain development progresses, the number of pathways enriched among upregulated genes in the LG increases, and the number of pathways enriched among downregulated genes decreases. During the cell division stage, genes involved in fatty acid metabolism pathways and plant hormone signaling pathways involved in cell wall synthesis are highly expressed. At the late stage of grain filling, genes involved in metabolic pathways, such as starch, carbohydrate, and sugar metabolism pathways are highly expressed (Figure 3). The continuous increase is consistent with the observed growth pattern, and it is speculated that these pathways contain genes related to grain size.

The construction of a gene coexpression network showed that the 200 genes in the pink module were significantly related to traits, such as grain weight. The expression of genes in this module is associated with the phenotypic differences in grain length and grain width between the two materials. The most significant item of gene GO enrichment in the pink module is nutrient storage activity, and the most significant pathway for KEGG enrichment is sugar metabolism, indicating that the formation of the final grain weight of LG is related to sugar transport and dry matter accumulation. The expression patterns of the two materials LG and SG diverged beginning at 15 DAF, which means that the difference in gene expression patterns of the two materials was mainly at the grain filling stage. In addition, the 12 candidate genes selected were all differentially expressed at 8 DAF and 15 DAF except HORVU7Hr1G006280. It is speculated that the remaining 11 candidate genes are continuously differentially regulated during the transition from cell division to grain filling.

HORVU6Hr1G055960 is annotated as a SWEET family intercellular sugar transporter. OsSWEET11 and OsSWEET15 play important roles in rice grain filling. During filling, a large amount of sucrose is transported to the developing caryopsis to provide nutrients and dry matter synthesis substrate for the cells. At the same time, sucrose is the carbon skeleton of cell wall starch biosynthesis (Yang et al., 2018). In the early stages of legume seed development, cell wall invertase decomposes incoming sucrose to produce hexose. These hexoses are thought to stimulate mitotic activity to increase cell numbers (Weber et al., 2005). The *ossweet11* mutant exhibits incomplete filling, shrunken grains, and a significant decrease in TGW. Cytological studies have found that the inhibition of OsSWEET11 and OsSWEET15 transporters can lead to the export of sucrose to the endosperm and the accumulation of starch in peripheral cells. *ZmSWEET4c* is highly expressed in maize from 10 DAF to 17 DAF. The starch content of the mature *zmsweet4c* mutant was approximately 10 times lower than that of the wild-type, and the TGW was also significantly reduced. In mutant seeds, the abundance of *ZmSWEET4*c transcripts is also greatly reduced (Sosso et al., 2015). The positive clones of yeast hexose transport mutant containing the HORVU6Hr1G055960 can grow on the medium containing glucan, providing that it can transport glucose (unpublished data). Compared to the rice and maize, we hypothesis that this gene was participated in the transport of the hexoses during grain filling and thus resulted in the big grain.

HORVU1Hr1G061160 is predicted to encode PPDK. PPDK plays a role in the biosynthesis of phosphoenolpyruvate to pyruvate and is highly expressed during grain development. Its homolog in rice, *FLO4/OsPPDK*, encodes pyruvate phosphate dikinase. The T-DNA insertion mutant *flo4* has powdery endosperm, but the outer endosperm is normal. Compared with the wild-type, the mutant has approximately 6% lower grain weight, a similar total amount of starch, and slightly higher total protein content (Kang et al., 2005).

HORVU7Hr1G020860, HORVU7Hr1G020880, and HORVU0Hr1G029210 encode ENOD proteins. The spatiotemporal expression patterns of *ENOD* genes during root nodule symbiosis in legume plants indicate that ENODs are involved in the interaction between rhizobia and root epidermal cells, as well as in cell differentiation and cell wall formation (Scheres et al., 1990). In pea and broad bean root epidermal cells, the ENOD5 protein was characterized as an arabinogalactan GPI membrane anchor and anchor point. Because the arabinogalactan-modified GPI membrane anchor and anchor point plays roles in cell signaling, cell differentiation, organization development, and signal transduction, it is inferred that ENOD5 is also involved in these physiological processes (Govers et al., 1991; Reddy et al., 1998; Varkonyi-Gasic and White, 2002). Frühling et al. (2000) found that the *ENOD12* gene in peas and alfalfa encodes a proline-rich protein, which is characterized by the repetition of the PPX_3_ pentapeptide in different quantities before the signal peptide. The PPX_3_ pentapeptide repeat sequence defines a class of structural cell wall proteins known as hydroxyproline-rich glycoproteins (HRGPs; Showalter, 1993). Therefore, the ENOD12 protein is a structural component of the cell wall in root nodules. As mentioned above, HORVU7Hr1G020860, HORVU7Hr1G020880, and HORVU0Hr1G029210 are significantly upregulated in the cell division stage of LG. As ENODs are known to be involved in cell wall synthesis, cell differentiation, and other physiological processes in soybean, pea, alfalfa, and other plants (Showalter, 1993), it is speculated that HORVU7Hr1G020860, HORVU7Hr1G020880, and HORVU0Hr1G029210 are involved in cell division and cell wall synthesis during the cell division stage of barley and are finally reflected in the extension of grain length.

HORVU1Hr1G088510 encodes MPK family members of mitogen-activated protein kinases, and the function of OsMPK7, its homolog in rice, is still unclear. Multiple members of the MPK family can regulate grain size. The *small grain 1* (*smg1*) mutant produces small and short grains due to a decrease in the number of glume cells (Duan et al., 2014). *SMG1* encodes MAPK kinase (OsMKK4), and its mutants, *small grain 2-1(smg2-1)*/*osmkkk10, smg1-1/osmkk4*, and *dwarf and small grain1(dsg1)/osmapk6*, are smaller than wild-type due to a decrease in the number of cells in the glume. OsMKKK10 phosphorylates and activates OsMKK4 and OsMAPK6 in turn, and OsMAPK6 activity is positively correlated with grain size. If OsMPK6 expression is inhibited, rice panicle grains became denser and smaller (Xu et al., 2018).

HORVU5Hr1G080540 encodes the E3 ubiquitin ligase, which is the ortholog of the E3 ubiquitin ligase BB in wheat. Not only does BB negatively regulate grain size in wheat, but DA1, DA2, and BB act synergistically to inhibit seed growth in *Arabidopsis* (Disch et al., 2006; Xia et al., 2013). In rice and wheat, BB restricts grain size by limiting the duration of the cell proliferation stage rather than the growth rate. BB is involved in regulating grain size in many plants, and its function is relatively conserved. Candidate genes enrich the genetic resources of molecular breeding. In addition to the seven candidate genes mentioned above, the orthologs of the other five genes have not been reported, and their functions are not yet known.

## Data Availability Statement

The datasets presented in this study can be found in online repositories. The names of the repository/repositories and accession number(s) can be found at: https://www.ncbi.nlm.nih.gov/, SRP344987.

## Author Contributions

JX and YS conceived and designed the research. DK performed the experiments. JX analyzed the transcriptome data. LW, HW, EY, XL, and TC assisted in the experiments and discussed the results. DK and JX wrote the manuscript. YS revised the manuscript. All authors contributed to the article and approved the submitted version.

## Funding

This work was supported by the Second Tibetan Plateau Scientific Expedition and Research Program (STEP; 2019QZKK0303), National Natural Science Foundation of China (General Program 32171958), and the Qinghai Province Natural Science Foundation (2020-ZJ-908).

## Conflict of Interest

The authors declare that the research was conducted in the absence of any commercial or financial relationships that could be construed as a potential conflict of interest.

## Publisher’s Note

All claims expressed in this article are solely those of the authors and do not necessarily represent those of their affiliated organizations, or those of the publisher, the editors and the reviewers. Any product that may be evaluated in this article, or claim that may be made by its manufacturer, is not guaranteed or endorsed by the publisher.

## Footnotes

^1^ http://www.geneontology.org/
