# Supplementary figures and images for: Combined RNA-seq and Phenotype Analysis Reveals a Potential Molecular Mechanism of the Difference in Grain Size of Naked Barley From the Qinghai–Tibetan Plateau
Source: Front Plant Sci. 2022 Feb 2;13:822607. doi: 10.3389/fpls.2022.822607 (PMC8847792; doi:10.3389/fpls.2022.822607)

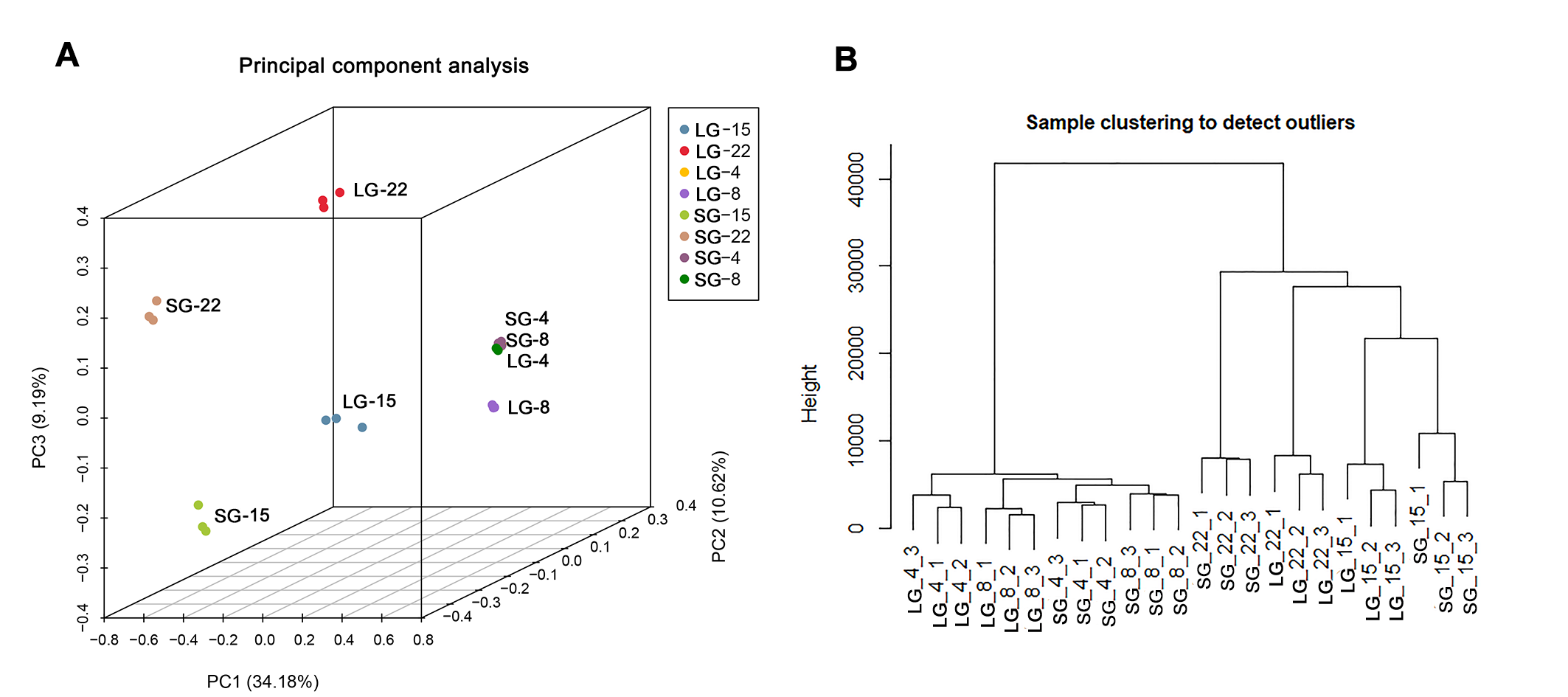

Supplement: Supplementary file 2 [file Image_1.TIF]

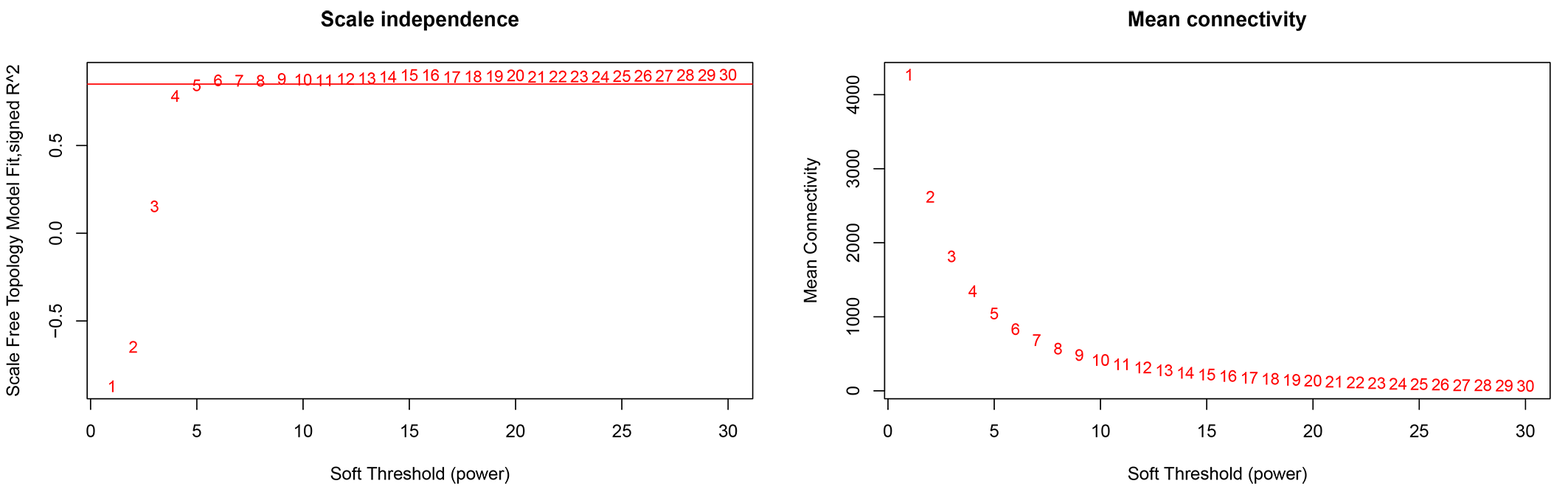

Supplement: Supplementary file 3 [file Image_2.TIF]
